# Nonoperative treatment of muscle injuries - recommendations from the GOTS expert meeting

**DOI:** 10.1186/s40634-018-0139-3

**Published:** 2018-06-22

**Authors:** T. Hotfiel, R. Seil, W. Bily, W. Bloch, A. Gokeler, R. M. Krifter, F. Mayer, P. Ueblacker, L. Weisskopf, M. Engelhardt

**Affiliations:** 1 Department of Orthopaedic Surgery, Friedrich-Alexander-University Erlangen-Nuremberg, Rathsbergerstraße 57, D-91054 Erlangen, Germany; 2 Department of Orthopaedic Surgery, Clinique d’Eich - Centre Hospitalier de Luxembourg, Luxembourg, Luxembourg; 3 Sports Medicine Research Laboratory, Luxembourg Institute of Health, Luxembourg, Luxembourg; 4 Department of Physical Medicine and Rehabilitation, Wilhelminenspital, Vienna, Austria; 5 Department of Molecular and Cellular Sports Medicine, Institute of Cardiovascular Research and Sports Medicine, German Sport University Cologne, Cologne, Germany; 6 Luxembourg Institute of Research in Orthopedics, Sports Medicine and Science, Luxembourg City, Luxembourg; 7 Exercise Science and Neuroscience, Department Exercise & Health Faculty of Science, Paderborn University, Paderborn, Germany; 8 ORTHOFOCUS-Orthopedic Competence Center, Graz-Salzburg, Austria; 9 Outpatient Clinic Potsdam, Sports Medicine & Sports Orthopaedics, University of Potsdam, Potsdam, Germany; 10 MW Center of Orthopedics and Sports Medicine, Munich, Germany; 11 Altius Swiss Sportmed Center, Rheinfelden, Switzerland; 12 Department of Trauma and Orthopedic Surgery, Klinikum Osnabrück, Osnabrück, Germany

**Keywords:** Muscle injuries, Muscle strain, Treatment, PRICE, Injection, PRP, Actovegin, Sports injuries, Consensus statement

## Abstract

**Background:**

Muscle injuries are some of the most common injuries in sports; they have a high recurrence rate and can result in the loss of ability to participate in training or competition. In clinical practice, a wide variety of treatment strategies are commonly applied. However, a limited amount of evidence-based data exists, and most therapeutic approaches are solely based on “best practice”. Thus, there is a need for consensus to provide strategies and recommendations for the treatment of muscle injuries.

**Methods:**

The 2016 GOTS Expert Meeting, initiated by the German-Austrian-Swiss Society for Orthopaedic Traumatologic Sports Medicine (GOTS), focused on the topic of muscle and tendon injuries and was held in Spreewald/Berlin, Germany. The committee was composed of twenty-two medical specialists. Nine of them were delegated to a subcommittee focusing on the nonoperative treatment of muscle injuries. The recommendations and statements that were developed were reviewed by the entire consensus committee and voted on by the members.

**Results:**

The committee reached a consensus on the utility and effectiveness of the management of muscle injuries. Main results: the “PRICE” principle to target the first inflammatory response is one of the most relevant steps in the treatment of muscle injuries. Haematoma aspiration may be considered in the early stages after injury. There is presently no clear evidence that intramuscular injections are of use in the treatment of muscle injuries. The ingestion of non-steroidal anti-inflammatory drugs (NSAIDs) should be regarded critically because there is currently no hard evidence to support their use, although they are appropriate in exceptional cases.

**Conclusions:**

The present work provides a structured overview of the various nonoperative treatment strategies of muscle injuries and evaluates their effectiveness with respect to the existing scientific evidence and clinical expertise in the context of basic science on the healing process of muscle injuries. The committee agreed that there is a compelling need for further studies, including high-quality randomized investigations to completely evaluate the effectiveness of the existing therapeutic approaches. The given recommendations may be updated and adjusted as further evidence will be generated.

## Review

## Background

Muscle injuries are frequently observed in both recreational and professional sports and are some of the most common sports injuries, accounting for up to 10–55% of all injuries (Best and Hunter 2000; Huard et al. 2002; Jarvinen et al. 2005). In professional football, muscle injuries are responsible for almost one third of all time-loss injuries and a team consisting of 25 players can expect nearly 15–18 injuries in a single season (Ekstrand et al. 2011). Respecting track and field athletes, during the IAAF World Championships between 2007 and 2015, 40.9% of all cases were diagnosed as muscle injuries and nearly 58% were assessed as time-loss injuries (Edouard et al. 2016). A great number of athletes will develop recurrence, a leading cause of longer rehabilitation time and time loss in sports (Ekstrand et al. 2011). At present, a wide variety of treatment strategies have been reported (Hotfiel et al. 2016b; Orchard et al. 2008; Reurink et al. 2012). Despite the high prevalence and the well-known, consequences of muscle injuries, many treatment strategies are based solely on expert-opinion or “best practice”. There is only limited evidence supporting even the commonly applied therapeutic strategies (Orchard et al. 2008; Reurink et al. 2012; Ueblacker et al. 2016). Moreover, diagnosis and classification of muscle injuries are not uniformly accepted (Mueller-Wohlfahrt et al. 2013). Hence, there is a need for a consensus to provide recommendations for the treatment of muscle injuries that must be based on the existing literature and on the fundamental principles of the healing and regeneration processes of muscle injuries. To unite the often contradictory approaches, the GOTS (German-Austrian-Swiss Society for Orthopaedic Traumatologic Sports Medicine) Expert Meeting was initiated.

The overall goal of the GOTS expert meeting was to provide guidance and recommendations for the initial on-field as well as advanced conservative therapy of muscle injuries and to suggest future directions for research regarding the conservative treatment of muscle injuries.

## Methods

**Fig. 1:**
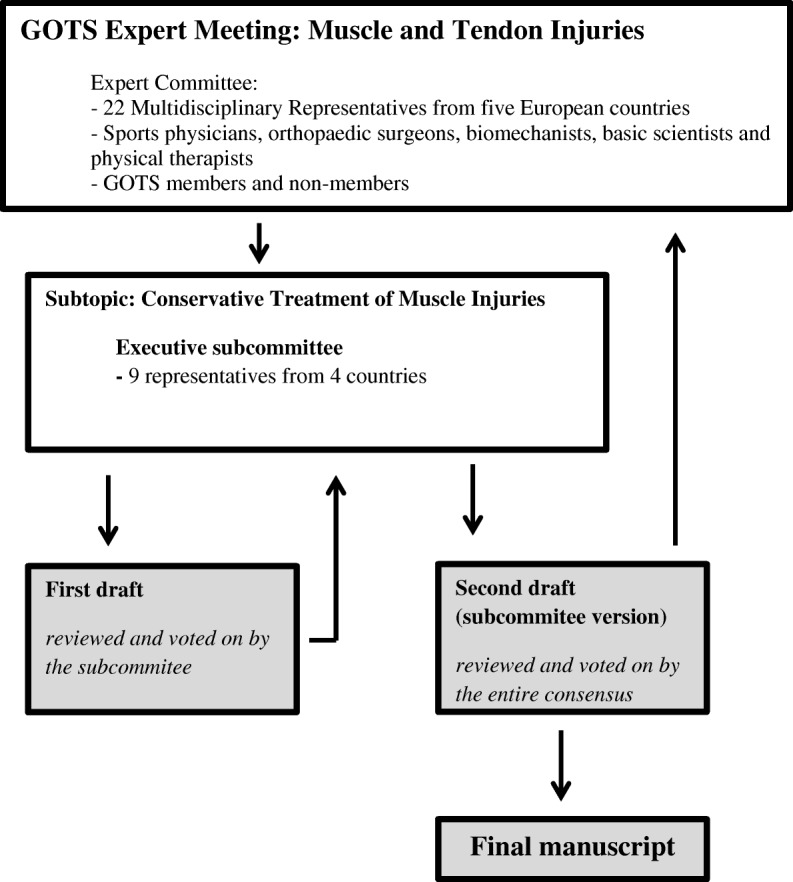
Flow diagram illustrating the organization and passage of the manuscript through the 2016 GOTS Expert Meeting

The biennial GOTS Expert Meeting was initiated in 2010 by the GOTS. The 2016 Expert Meeting focused on the topic of muscle and tendon injuries and was held in Spreewald/Berlin, Germany, in May of 2016. An international committee comprised of sports physicians, orthopaedic surgeons, biomechanists, basic scientists and physical therapists took part in the meeting. Various sub-committees (*n* = 10) were formed that focused on muscle, tendon and connective tissue in regard of injury mechanisms, classification and epidemiology, anatomy and healing mechanisms, clinical and radiological spectrum of diagnostics, conservative and operative treatment as well as rehabilitation and prevention. For the purpose of this manuscript the work done by a subcommittee consisting of 9 representatives from 4 countries that focused on the conservative treatment of muscle injuries is highlighted. Prior to the meeting, this committee reviewed the existing literature on the treatment of muscle injuries, including original research reports, systematic and non-systematic reviews, book chapters and case reports. The subcommittee worked in groups prior to presenting it to the entire committee. Consensus was reached on inclusion of level 1–2 evidence. If level 1–2 evidence was lacking the committee reverted to present recommendations based on a consensus discussion after reviewing available literature and/or best clinical practice. The developed recommendations and statements were reviewed by the entire consensus committee and voted on by the members. The subsequent draft manuscript provided by the subcommittee was distributed for feedback from the entire committee and was then revised and discussed again with input according to the committee’s suggestions (Fig. 1).

## Results

### Primary care and initial treatment

**Fig. 2:**
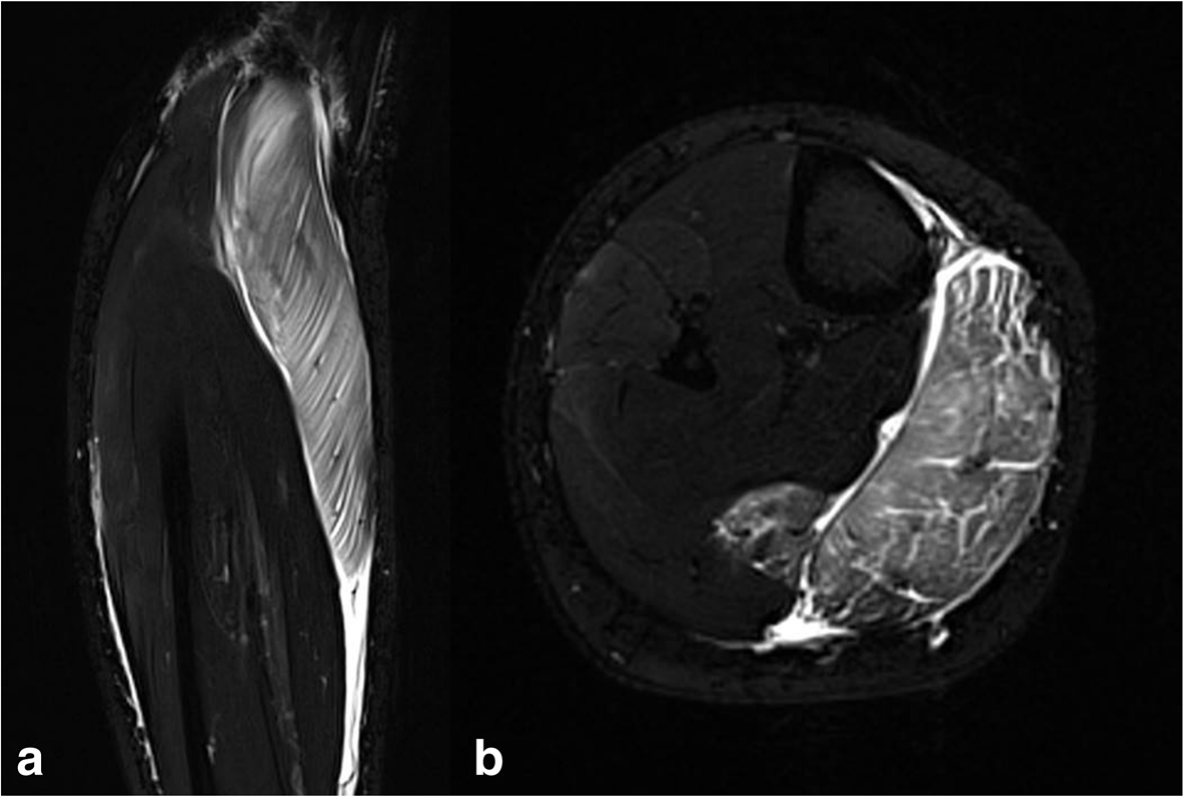
Indirect ultrastructural muscle injury (Type 1B: Delayed-onset muscle soreness (DOMS)). The MRI demonstrates a widespread intramuscular oedema corresponding to a raise in T2 signal intensity. **a**: Axial T2-weighted TIRM (Turbo-Inversion Recovery-Magnitude) sequence (total acquisition time, 3:31 min; inversion time, 260 ms; echo time, 69 ms; repetition time, 5120 ms; flip angle, 145°; resolution, 0.8 × 0.8 × 4.0 mm). **b**: coronal T2-weighted TIRM sequence (total acquisition time, 3:42 min; inversion time, 260 ms; echo time, 68 ms; repetition time, 5120 ms; flip angle, 180°; resolution, 0.9 × 0.9 × 4.0 mm). Published by kind permission of the Department of Radiology, University Hospital Erlangen

Although the initial treatment is suggested to be one of the most relevant steps in the management of muscle injuries (Delos et al. 2013; Hotfiel et al. 2016b; Jarvinen et al. 2005; Ueblacker et al. 2016), evidence to substantiate this is lacking. Primary treatment has been widely advocated to include the Protection, Rest, Ice (Cold), Compression and Elevation (the acronym “PRICE”), despite the lack of any high-quality studies to support its evidence (Hotfiel et al. 2016b; Ueblacker et al. 2016). The “PRICE” principle is commonly recommended for the initial treatment of most sports injuries (Bleakley et al. 2004; Bleakley et al. 2012; Collins 2008; van den Bekerom et al. 2012). The primary therapy target is to minimize intramuscular bleeding to the injured area and to further reduce pain, intramuscular oedema and formation of scar tissue. Inadequate initial treatment may also increase the risk of overestimating the injury during subsequent imaging owing to the presence of haematoma or oedema, which correspond to an raise in T2 signal intensity in MRI (Hotfiel et al. 2017b; Kellermann et al. 2017a; Ueblacker et al. 2016) (Fig. 2).

#### Protection/Rest

Both restricting activity and immobilization should prevent hamper healing of the injury. This protects the ruptured muscle ends from further retraction, potentially reinforcing the intramuscular gap (Jarvinen et al. 2005). It is recommended as best clinical practice that the affected limb should be immobilized, placing the athlete in a position that relieves stress on the affected muscle fibres (Ueblacker et al. 2016).

#### Ice

Generally, cryotherapy is applied for soft tissue sports injuries (Bleakley et al. 2004; Collins 2008; Hubbard and Denegar 2004). The ideal application involves an ice-soaked sponge or ice pack wrapped into a towel (Ueblacker et al. 2016). The application of ice leads to vasoconstriction and reduces the local muscular blood flow by approximately 50% after 10 min (Thorsson 2001); this leads to a decrease in swelling and initial bleeding. The early use of cryotherapy has also been found to decrease both inflammatory reactions and oedema and to accelerate early regeneration in experimental settings (Deal et al. 2002; Hurme et al. 1991; Swenson et al. 1996). Another effect is the reduction of pain by increasing threshold levels in the free nerve endings and at synapses by raising nerve conduction latency to promote analgesia (van den Bekerom et al. 2012). Ice spray can also be applied, particularly for pain relief. Generally, direct contact between the applied ice and the athlete’s skin should to be avoided to prevent blistering or necrosis of the skin.

In terms of compression, attention should be paid to correct application; if used properly, it can be regarded as the most effective method in the initial therapy for structural injuries by encouraging haemostasis via reducing intramuscular bleeding. Initially, the compression load chosen at the region of injury should be as high as possible to target the arterial vessels responsible for pronounced bleeding. Ideally, elastic compression bandages should be available in different styles to adapt the size to the region of injury. Following the primary haemostasis, the compression load can be reduced after 20–30 min. It must be noted that continuous, moderate compression should be applied on the entire extremity to stimulate lymphatic flow and thus promote the reduction of further swelling. In the case of high-grade injuries (subtotal/total muscle ruptures), a potentially large amount of intramuscular bleeding should be expected. Consequently, the applied pressure load must be checked regularly for imminent compartment syndrome (Hotfiel et al. 2016b).

#### Elevation

Based on physiological principles, raising the lower limb above the heart level leads to a decrease in intravascular hydrostatic pressure, thereby limiting bleeding and the accumulation of interstitial fluid (Jarvinen et al. 2005).

Finally, the initial (on-field) therapy is one of the most important steps in the treatment of muscle injuries and should be provided as soon as possible. In the ideal case, constant observation of the players’ action on the field may aide the physician to assess the mechanism of injury. Subsequent to the injury event, the essential first steps of treatment are required. In some cases, a treatment in the performance area or on a side-line must be permitted by the referee or security guard. Hence, the subsequent initial on-field or sideline therapy (according to the referee) may display the ideal condition to limit initial intramuscular bleeding and further inflammation. Detailed questioning about complaints and a comprehensive examination should be avoided to not waste valuable time which is crucial to the first treatment steps.

*The initial therapy is one of the most relevant steps in the treatment of muscle injuries. PRICE therapy should be applied after the injury event to obstruct the first inflammatory response by preventing the formation of intramuscular haematoma and interstitial oedema.*

**Fig. 3:**
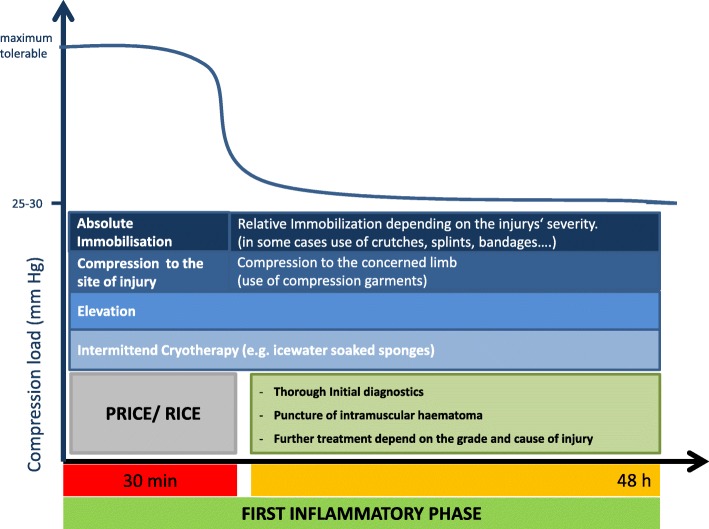
Illustration of the treatment algorithm for the initial therapy during the first inflammatory phase (0–48 h after the injury event)

After the initial treatment and a thorough diagnosis that aims to evaluate the injury’s severity in detail (Fig. 3), many therapeutic interventions can be considered. Moreover, depending on the injury’s severity, a decision must be made about the re-mobilization process. Although the term “early mobilization” is nowadays often used, it was first described in 1954 (Hamilton 2012). However, there is no clear-cut definition in the treatment of muscle injuries. No studies have been conducted to investigate clinical outcome in athletes who were allocated to different and clearly defined times of immobilization after the injury event. Based on both the authors’ experiences and the recommendations of the existing literature, the committee suggests a relative immobilization during the first 3–5 days after injury for structural injuries depending on the type and severity of the specific injury (Heiderscheit et al. 2010; Hotfiel et al. 2016b). Due to the principles of pathophysiological healing phases, this time corresponds to the first inflammatory phase, in which limiting its expression must be given priority (Mendiguchia and Brughelli 2011; Sherry and Best 2004).

The “restricted” approach of 3–5 days of immobilization followed by an early mobilization can be further supported based on knowledge of early scar tissue formation, which has been considered to have an essential role in temporary bridging of the rupture zone. This early scar tissue provides an extracellular matrix that enables the satellite cells to migrate (Jarvinen et al. 2013; Jarvinen et al. 2005). The improvement in the regeneration and integrity of myofibres due to early mobilization is still being critically discussed; however, the up-regulation of satellite cells and their formation into myoblasts begins a few days after injury (Jarvinen et al. 2013; Karalaki et al. 2009).

*In structural muscle injures (grade IIIa-IV) (Mueller-Wohlfahrt* et al. 2013*), a relative immobilization of 3–5 days must be seen as appropriate. Preferentially, a clinical follow-up examination that includes imaging (ultrasound) should be taken into account prior to further decision-making about mobilization to exclude any delayed onset manifestation of intramuscular damages.*

### Interventional treatment strategies

#### Aspiration of intramuscular haematoma

**Fig. 4:**
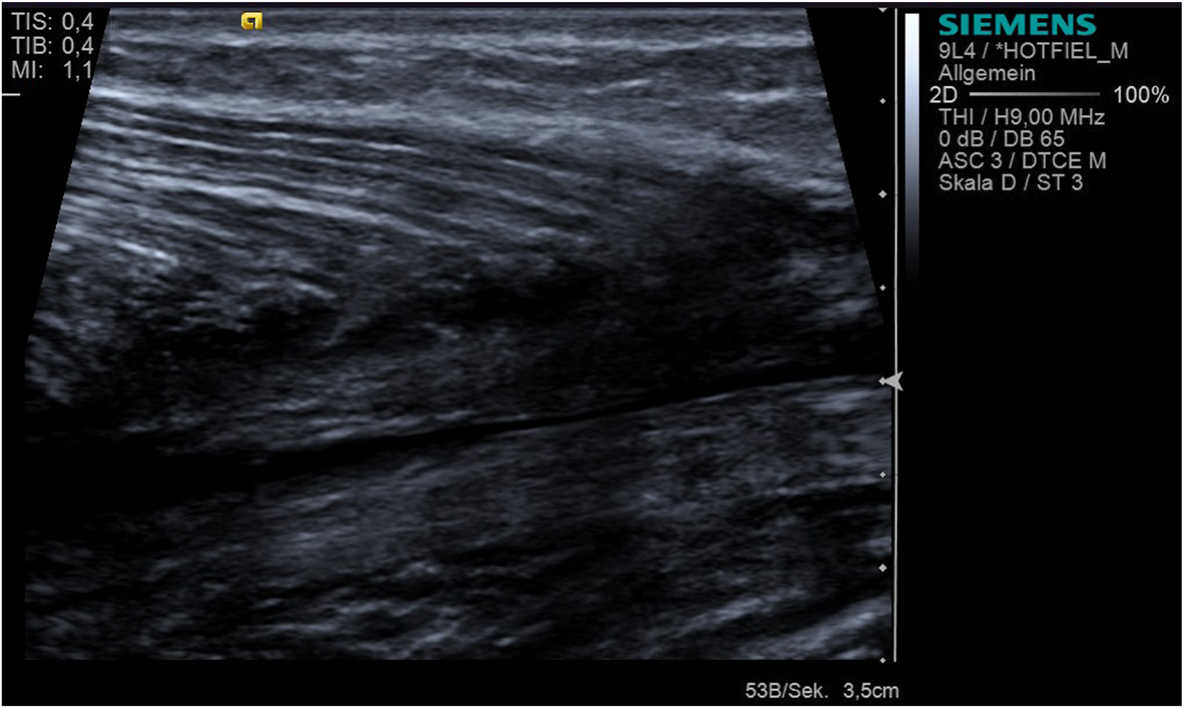
Longitudinal ultrasound scan of a dorsal calf demonstrating a clearly detectable haematoma (12 h after injury) at the medial gastrocnemius muscle

**Fig. 5:**
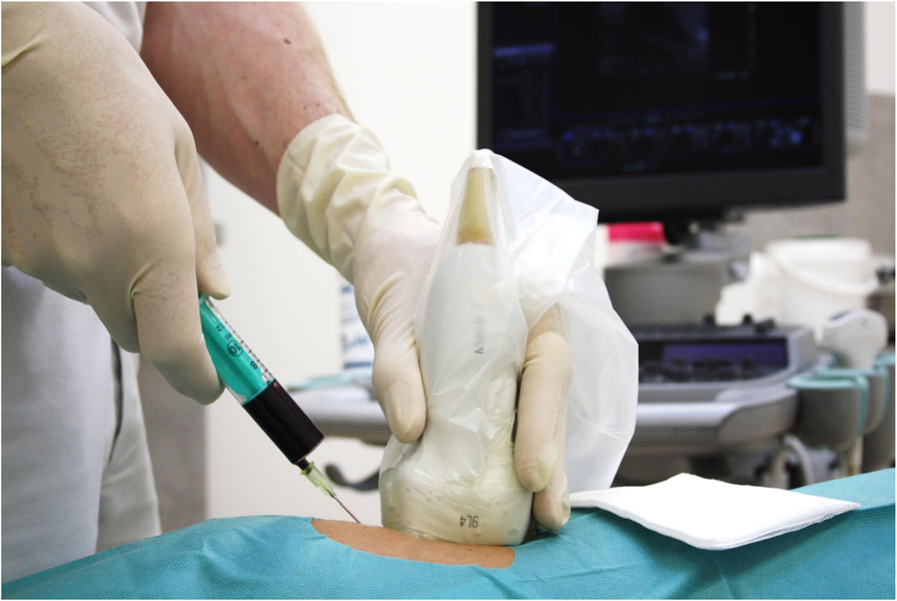
Ultrasound guided puncture of a haematic lesion in context of a structural muscle injury of the rectus femoris muscle

Structural muscle injuries are commonly represented by a tear or disorganization of the muscle fibre and by damage to blood vessels that results in intramuscular haematoma (Balius et al. 2014; Courthaliac et al. 2005; Hotfiel et al. 2016a; Kellermann et al. 2017a; Kellermann et al. 2017b; Peetrons 2002) (Fig. 4). The accumulation of intramuscular blood cells may contribute to a further migration of inflammatory cells, thereby reinforcing the first inflammatory phases. It is well known that an optimized regeneration phase requires a previous absorption of emitted blood components and cell debris (Jarvinen et al. 2013; Sciorati et al. 2016). To speed up this process and to limit subsequent formation of scar tissue on the injury site, aspiration of intramuscular fluid and haematoma can be considered. Ultrasound has been established as a valuable imaging tool to evaluate morphological aspects of a present haematoma or to guide the needle aspiration (Guillodo et al. 2011; Hotfiel et al. 2017a; Hotfiel et al. 2017b; McCarthy et al. 2016). It is commonly accepted, that the assessment of hematoma remains challenging in the early stages after injury as hematoma may develop delayed and acute hemorrhages are difficult to separate from healthy surrounding tissue (Draghi et al. 2013; Peetrons 2002). Hence we recommend follow-up examinations at 48 h and during the decision-making process about the time of mobilization to exclude any delayed onset manifestation of intramuscular hematoma (Drews et al. 2017; Peetrons 2002). The technique can be performed through two different approaches: guiding the puncture via previous scanning and determining the optimal puncture site, or guiding the needle during the examination in real-time (Fig. 5). The puncture should be performed promptly after the appearance of an intramuscular haematoma as haematomas mostly begin to spread outside of the region of injury (Peetrons 2002), and the technique could be hindered by coagulated blood coagula that begin to organize after several days. However, no studies have investigated the therapeutic effectiveness of intramuscular puncture with respect to healing, the return to sports or the re-injury rate. Thus, the technique of intramuscular puncture is solely based on expert opinions and pathophysiological foundations. When considering puncturing a haematoma, it is very important to use aseptic techniques and to perform a compression treatment controlled by clinical and ultrasound checks performed after 2 and 5 days.

*The puncture of (sero) haematic lesions can be performed to optimize the regeneration and healing phases of structural muscle injuries depending upon both the localization and age of the existing haematoma. The technique is restricted in the case of diffuse intramuscular bleeding, as this morphologic circumstance makes it difficult to aspirate the entirety of the blood accumulation.*

#### The role of injection therapy

Injections with various substances are frequently used in the clinical setting. A high number of diverse pharmaceutical agents are used, yet there is limited evidence supporting this. The most commonly used agents are listed below:

##### Corticosteroid injections

The local and systemic application of corticosteroids is not recommended in the treatment of muscle injuries. There is one existing study showing the positive therapeutic effects of corticosteroid injections in muscle injuries; however, it uses a poor methodological approach and a small sample size (*n* = 6, no randomization) (Levine et al. 2000). The intramuscular presence of corticosteroids is associated with several adverse effects regarding the pathophysiological healing phases of muscle injuries. Corticosteroids hinder the absorption of intramuscular haematoma and simultaneously enlarge intramuscular necrosis and the risk of infection in the area of injury (Beiner et al. 1999; Järvinen and Sorvari 1992). Moreover, their application has been considered to prolong the rehabilitation phases and has also been shown to have negative effects on the neuromuscular response. A systematic review showed moderate to strong evidence of the myotoxic effects of corticosteroids, particularly for combined use with local anaesthesia (Reurink et al. 2014b). As an exception, the use of corticosteroids may be appropriate in the case of chronic muscle injury. However, recommendations on the dosage, injection time or the exact corticosteroid composition cannot be treated for this case. The issue of chronic muscle injury has always been poorly understood, and the terminology has been inconsistent. Due to pathophysiological considerations, the chronic process could be associated with the autoinflammatory processes and a dysregulation of immune cells (Sciorati et al. 2016). Further studies are required to determine whether the use of corticosteroids could help limit the intramuscular autoimmune dysregulation in this exceptional case.

##### Actovegin®

Actovegin® (Takeda Austria GmbH, Linz, Austria) is a purified, deproteinized haemodialysate extracted from bovine-origin blood by ultrafiltration. Actovegin is intended to have an important role for ergogenic properties and muscle tissue metabolism. In recent years, the use of Actovegin® in the treatment of muscle injuries has been controversial. A recent study analysing the effects of Actovegin® on muscle tissue in vitro was able to demonstrate an Actovegin®-dependent myoblast stimulation and an increased myoblast fusion through the same means (Reichl et al. 2017). Those alterations are thought to be based on an improved satellite cell activation - an essential initial step in the healing process of muscle injuries (Karalaki et al. 2009). On the basis of the above-mentioned findings, the use of Actovegin® may be appropriate between day three and day ten after injury. A study investigating the mitochondrial respiratory capacity in human skeletal muscle fibres acutely exposed to Actovegin® demonstrated an increase in mitochondrial oxidative function, thereby showing Actovegin® to have potential ergogenic properties (Sondergard et al. 2016). In terms of potential ergogenic effects in athletes, the authors highlight the need to investigate whether Actovegin® should be included on the World Anti-Doping Agency’s active list (Sondergard et al. 2016). However, few available studies focus on the clinical use of Actovegin® in human skeletal muscle injuries, and evidence-based data are missing. A clinical controlled study reported an improved regeneration and shorter time loss of sports for grade (*n* = 3), but a control group was missing (Lee et al. 2011). On the current World Anti-Doping Prohibited List, its intravenous use is forbidden. It should be noted that the approval of Actovegin® is restricted in some countries.

##### Platelet-rich plasma (PRP)

PRP injection has become a popular intervention in orthopaedics and sports medicine. In recent years, PRP has gained increasing attention in the treatment of diverse musculoskeletal disorders to accelerate tissue healing, including acute or chronic tendon disorders, plantar fasciitis, ligament or muscle injuries, and even the process of osteoarthritis. PRP as an autologous product is acquired from a sample of the athletes’ blood and centrifuged to obtain a large number of platelets in a volume of plasma (Alessandrino and Balconi 2013; Andia and Maffulli 2015). Platelets are intended to release diverse proteins and cytokines, thereby enhancing the healing and regeneration responses of the injured tissue. In addition to a wide variety of substances that have recently been described as ingredients, some fundamental proteins have been found to be released; these include platelet derived growth factors (PDGFs), transforming growth factor–β (TGF-β), vascular endothelial growth factor (VEGF), epidermal growth factor (EGF), and adhesive proteins such as fibrin, fibronectin, and vitronectin (Jeong et al. 2014). In the past few years, PRP has frequently been used in the treatment of muscle injuries in the clinical setting. Because its performance-enhancing effects could not be confirmed, PRP was removed from the World Anti-Doping Prohibited List in 2012. However, the selective injection of separated growth factors is indeed a prohibited method according to the current World Anti-Doping Prohibited List of 2018.

Several in vivo laboratory studies suggested that PRP could induce either an upregulation of satellite cell activation or the myogenic differentiation into myoblasts and myofibres, thereby accelerating the recovery of damaged muscle tissue (Dimauro et al. 2014). However, there has been no clear-cut evidence to support this hypothesis to date (Miroshnychenko et al. 2017).

There are many studies evaluating the therapeutic effectiveness of PRP in humans based on clinical outcome parameters. In general, most of the existing studies were unable to demonstrate therapeutic effectiveness due to the intramuscular application of PRP. A double-blind, placebo-controlled trial including 80 competitive and recreational athletes with acute hamstring muscle injuries could not show any therapeutic superiority of intramuscular PRP injections over placebo injections (Reurink et al. 2014a). A randomized, double-blind study involving 90 professional athletes with MRI-positive acute hamstring injuries indicated that there is no benefit of a single PRP injection with respect to return to play and the re-injury rate after 2 and 6 months (Hamilton et al. 2015). Existing systematic reviews have consistently concluded that there is currently no evidence to support the use of PRP in the treatment of muscle injuries (Andia and Maffulli 2015; Guillodo et al. 2015; Hamid et al. 2014; Moraes et al. 2014; Pas et al. 2015). A methodological weakness of the existing studies is that they involved different application protocols and PRP preparation techniques, with different growth factor concentrations (Kieb et al. 2017). Based on experimental studies, existing scientific gaps and technological barriers such as comparable protocols and preparation methods must be addressed to clarify the potential promising effects of PRP (Mosca and Rodeo 2015).

In view of their potential adverse effects, the presence of diverse fibrotic agents has recently been critically examined. TGF-β in particular has been observed to promote fibrosis during the regeneration phase (Evans 2013; Kelc et al. 2015; Terada et al. 2013). Some studies suggest that the combined application of PRP and anti-fibrotic agents could promote the healing process by limiting an excessive process of fibrosis (Evans 2013; Kelc et al. 2015; Terada et al. 2013). Further well-designed studies focusing on the combined application of PRP and anti-fibrotic agents must be implemented to evaluate the potential benefits of this approach.

*Presently, there is no clear evidence that intramuscular injections of PRP are efficacious in the treatment of muscle injuries. Thus, the use of PRP cannot be generally recommended for the treatment of muscle injuries.*

##### Local anaesthetics

The main function of local anaesthetics is to block sodium ion channels, which play an essential role at the membranes of motor endplates responsible for efferent neuro-transmission to develop neuromuscular forces. Moreover, sodium ion channels are located at free nerve endings that refer to afferent pain transmission. Many physicians who use local anaesthetics along the longitudinal fascia a muscle (Ueblacker et al. 2016) or solely in the centre of a lesion expect a decrease in muscle tightness, which should improve the regeneration process. However, no existing randomized trials have evaluated the clinical effects of local anaesthetic injections in the treatment of muscle injuries. There are some studies describing the myotoxic properties of local anaesthetics, but they are mostly based on animal models (Neal et al. 2016; Oz Gergin et al. 2015; Zink et al. 2002). A systematic review reported moderate to strong evidence of intramuscularly injected local anaesthetics induced myotoxic effects (Reurink et al. 2014b). The exact mechanisms of the cytotoxic effect on skeletal muscle cells are still not fully understood (Metterlein et al. 2015). The involvement of intracellular calcium homeostasis has been shown to have great importance (Metterlein et al. 2015; Zink et al. 2002).

*Injections of local anaesthetics should be used with caution and their use cannot be recommended for the treatment of muscle injuries in general. The comprehensive use of local anaesthetics in clinical practice should be critically revisited.*

### Oral medication - is there a justification for the use of NSAIDs?

The use of non-steroidal anti-inflammatory drugs (NSAIDs) is widely promoted by many practitioners in the routine treatment of muscle injuries, and it has been used from the 1960s up to the present day (Hamilton 2012). In clinical practice, the administration of NSAIDs in the early phase of an injury is often recommended, mostly during the first 2–3 days after injury. Despite the ongoing extensive use by many practitioners, there is currently a trend towards taking a critical perspective on NSAIDs. The potential of NSAIDs to influence skeletal healing has been extensively investigated, with divergent outcomes. Some studies suggest an association between NSAIDs and an increased development of fibrosis during the healing process, resulting in an increased presence of scar tissue (Paoloni et al. 2009; Ziltener et al. 2010). However, there is some evidence to support their use in the context of satellite cell activation, which is likely to have a key function in the healing of muscle injuries (Hurme et al. 1991; Jarvinen et al. 2005; Sciorati et al. 2016). A placebo-controlled study investigating the satellite cell response on eccentric muscle contractions provoked by intense neuromuscular electrical stimulation (1200 mg Ibuprofen/d, 48–96 h post-intervention) observed beneficial effects with regard to enhanced satellite cell proliferation and accelerated repair of myofibres in the later stages of regeneration (Mackey et al. 2016). However, it is important to consider that neuromuscular electrical stimulation-induced muscle damage does not represent the conditions of physiological loading or even structural muscle tears (Mackey et al. 2016). From the clinical perspective, due to alterations in the perception of pain and sensorimotor control of the affected muscle, NSAIDs could cover protective pain signals. Following these masked pain signals, the athlete could be misled towards a rapid exercise load during the rehabilitation process, thereby increasing the risk of re-injury. The off-label use of indomethacin, provided in the context of myositis ossificans (MO), must be kept separate. Myositis ossificans, which is considered to be a heterotopic, non-neoplastic bone or cartilage formation reflecting a complication of structural muscle injuries (Alessandrino and Balconi 2013; Delos et al. 2013), is mostly associated with a history of intramuscular haematoma. Ultrasound has become a reliable tool to expose the presence of MO (Alessandrino and Balconi 2013). If signs of a beginning MO (calcified shadow signs at the periphery of the lesion in ultrasound imaging (Peetrons 2002; Ueblacker et al. 2016)) or injuries which may be at high-risk to develop a MO are present (diffuse intramuscular hemorrhage, severe muscle contusions in the vastus intermedius or soleus muscle (Peetrons 2002)), the ingestion of indomethacin for off-label use can be considered. However, the optimal prevention and treatment of MO is still unknown (Delos et al. 2013).

*There is currently no justification to support the use of NSAIDs in the treatment of muscle injuries. The general ingestion of NSAIDs should be regarded critically. The off-label use of indomethacin, provided in the context of preventing MO, can be appropriate in high-grade injuries showing signs of a beginning myositis ossification.*

### Physical therapy

Therapeutic ultrasound. Therapeutic ultrasound is a physical intervention frequently applied in the clinical setting. It uses a low frequency range adjusted between 0.8 and 3 MHz. It is referred to as a method of mechanical stimulation and energy transfer and has generally been attributed to an increase in blood flow and recovery, pain relief, anti-inflammatory action and tonus-modulating effects (Ebenbichler 2009). At present, the effects of therapeutic ultrasound on muscle injuries have been investigated only in animal models and in terms of their histopathologic and biomechanical aspects (Chongsatientam and Yimlamai 2016; Freitas et al. 2007; Montalti et al. 2013; Piedade et al. 2008; Vasquez et al. 2014). There are no evidence-based studies that have determined the clinical effects of therapeutic ultrasound on human muscle injuries. From the pathophysiological point of view, mechanical stimulation could lead to an increase in myoblast cell activity and an optimized revascularization. In terms of the application setting, we recommend the use of therapeutic ultrasound after the first inflammatory phase, as follows: dynamic pulsed ultrasound with intensity up to 1 W/cm^2^. Once a complete absorption of intramuscular hematoma is obtained (possibly controlled via diagnostic ultrasound prior to the upcoming intervention), the intensity can be increased up to 2 W/cm^2^. The treatment time should be adapted to the size of the lesion, ranging between 5 and 15 min.

*Due to experimental knowledge of the cellular effects of therapeutic ultrasound on muscle tissue, the use of therapeutic ultrasound should be seen as another potentially therapeutic modality in the treatment of muscle injuries. However, there is currently limited evidence that confirms its clinical benefits.*

## Future directions

The effectiveness of anti-fibrotic agents in the treatment of muscle injuries has been studied solely in animal models. These studies have focused on reducing the post-traumatic formation of fibrosis by improving the remodelling process (Evans 2013; Garg et al. 2015; Kelc et al. 2015; Terada et al. 2013). Approaches based on stem cell therapies, which describe the intralesional use of various progenitor cells—particularly in combination with extracellular scaffolds (Tissue Engineered Muscle Construct, TEMC)—are based on experimental approaches in animal models (Sicari et al. 2014). The application of acellular scaffolds is thought to optimize the rupture by improving the migration of satellite cells (Sicari et al. 2014). In view of the given anti-doping restrictions, which prohibit the injection of growth factors (Prohibited Substances S2) and the use of normal and modified genetic cells (Prohibited Methods M3), (WADA 2018) a comprehensive review of this topic has been excluded from this manuscript. It is still unknown to what degree Vitamin D contributes to the healing process or the occurrence of muscle injuries in the first place. Some studies support the potentially beneficial effects of using Vitamin D in muscle injuries; they show a correlation between Vitamin D and muscular function with respect to muscle strength and physical performance (Ceglia 2008; Hildebrand et al. 2016; Pfeifer et al. 2002). However, there is currently no scientific rationale for the comprehensive ingestion of Vitamin D. Thus, there is a need for future studies to investigate the potential beneficial effects of using Vitamin D in muscle injuries.

A limitation to the current scientific data is that the existing literature on the treatment of muscle injuries is solely based on relatively young athletes. Considering the demographic changes representing a growing number of older and active athletes, we call for future studies that investigate older athletes.

## Conclusions

The present work provides a structured overview of the diverse conservative treatment strategies of acute athletic muscle injuries and evaluates their effectiveness with respect to the existing scientific evidence and clinical expertise in the context of basic science on the healing process of muscle injuries. The committee agreed that there is a compelling need for further studies, including high-quality randomized investigations for a comprehensive evaluation pertaining the effectiveness of the existing therapeutic approaches. The given recommendations may be updated and adjusted.
